# The PERK signaling pathway as a marker of the unfolded protein response in patients with acute myeloid leukemia

**DOI:** 10.55730/1300-0144.6168

**Published:** 2025-10-08

**Authors:** Nergiz ERKUT, Turhan KÖKSAL, Selim DEMİR, Ahmet MENTEŞE, Özlen BALTA, Mehmet SÖNMEZ

**Affiliations:** 1 Division of Hematology, Department of Internal Medicine, Faculty of Medicine, Karadeniz Technical University, Trabzon, Turkiye; 2 Division of Nutrition and Dietetics, Faculty of Health Sciences, Karadeniz Technical University, Trabzon, Turkiye; 3 Division of Medical Biochemistry, Faculty of Medicine, Karadeniz Technical University, Trabzon, Turkiye

**Keywords:** Endoplasmic reticulum stress, PERK signaling pathway, ATF6 signaling pathway, hypoxia, apoptosis

## Abstract

**Background/aim:**

The protein kinase RNA-like endoplasmic reticulum kinase (PERK) pathway plays a critical role in preventing the accumulation of misfolded or unfolded proteins within the endoplasmic reticulum. In this study, the role of the PERK signaling pathway was evaluated in newly diagnosed, treatment-naïve patients with acute myeloid leukemia (AML).

**Materials and methods:**

Plasma levels of eukaryotic translation initiation factor 2-alpha kinase 3 (eIF2AK3), glucose-regulated protein 78 (GRP78), activating transcription factor 6 (ATF6), CCAAT/enhancer-binding protein homologous protein (CHOP), hypoxia-inducible factor-1 alpha (HIF-1α), and caspase 3 were measured by enzyme-linked immunosorbent assay in peripheral blood samples obtained from AML patients and healthy controls.

**Results:**

A total of 40 individuals were included, comprising 19 (47%) AML patients and 21 (53%) healthy controls. HIF-1α, eIF2AK3, GRP78, ATF6, CHOP, and caspase 3 levels were significantly higher in the AML group than in the control group (p = 0.019, 0.005, <0.001, 0.006, <0.001, and <0.001, respectively). No significant differences were observed in HIF-1α, GRP78, ATF6, CHOP, and caspase 3 levels between diagnosis and the 30th day of remission-induction therapy in the AML group, whereas a significant decrease was observed in eIF2AK3 levels (p = 0.049). At diagnosis, a strong positive correlation was found between GRP78 and CHOP levels (r = 0.740, p < 0.001), and a moderate positive correlation was detected between CHOP and caspase 3 levels (r = 0.514, p = 0.024) in the AML group. In the Cox regression analysis of the AML cohort, no statistically significant association was identified between overall survival and age, risk category, or biomarker levels (HIF-1α, eIF2AK3, GRP78, ATF6, CHOP, and caspase 3).

**Conclusion:**

PERK and ATF6 signaling pathways were activated in patients with AML. Targeting the unfolded protein response pathway may represent a promising therapeutic strategy for patients with AML.

## 1. Introduction

Acute myeloid leukemia (AML) is a biologically heterogeneous malignancy characterized by the uncontrolled proliferation of clonal hematopoietic cells [1]. The median age at diagnosis is 68 years [2]. The 5-year overall survival (OS) rate remains approximately 30% (SEER data base: Cancer Stat Facts: Leukemia - Acute Myeloid Leukemia). The etiology of AML involves hematologic disorders such as myelodysplastic syndrome and myeloproliferative neoplasms, as well as environmental and iatrogenic factors including smoking, exposure to benzene and its derivatives, chemotherapy, radiotherapy, ionizing radiation, congenital bone marrow failure syndromes, and germline mutations [3]. AML-associated somatic mutations progressively accumulate in hematopoietic stem and progenitor cells (HSPCs) with advancing age. These preleukemic conditions, termed clonal hematopoiesis of indeterminate potential and clonal cytopenia of undetermined significance, carry an increased risk of transformation to AML over time [4,5]. The identification of genetic drivers and advances in understanding disease biology have significantly contributed to risk stratification and therapeutic development in AML. However, due to the biological heterogeneity of AML, the underlying pathologic mechanisms remain incompletely understood [3].

The endoplasmic reticulum (ER) functions as a quality control organelle responsible for maintaining protein homeostasis. Endoplasmic reticulum stress (ERS) arises from the accumulation of unfolded or misfolded proteins within the ER, particularly under pathological conditions. In response to ERS, three distinct signaling pathways collectively known as the unfolded protein response (UPR) are activated to restore protein homeostasis [6]. These pathways include protein kinase RNA-like endoplasmic reticulum kinase (PERK), activating transcription factor 6 (ATF6), and inositol-requiring enzyme 1 alpha (IRE1α) [7–9]. Microenvironmental factors such as hypoxia, acidosis, nutrient deprivation, metabolic alterations, oncogenic stress, chemotherapy, and radiotherapy can induce ERS during tumor development [7]. Studies in patients with solid tumors and hematologic malignancies have demonstrated that ERS and UPR activation are linked to malignant transformation and disease progression [10]. This study investigated the role of the PERK signaling pathway in newly diagnosed, treatment-naïve patients with AML.

## 2. Materials and methods

### 2.1. Patients

The study included 19 newly diagnosed patients with AML and 21 healthy individuals who were recruited at the Department of Hematology, Faculty of Medicine, Karadeniz Technical University, between December 2022 and July 2023. Patients younger than 18 years, those with a history of prior treatment, coexisting hematologic or solid malignancies, or active infections were excluded from the study.

The diagnosis of AML was established based on morphological, immunophenotypic, and genetic findings in accordance with the World Health Organization (WHO) 5th edition classification criteria [1,11]. Patients diagnosed with AML received standard remission-induction therapy consisting of cytarabine (Ara-C) and idarubicin (the 7 + 3 regimen). In addition, patients did not receive FLT3 inhibitor therapy.

### 2.2. Blood samples and parameters

Blood samples were obtained from patients at diagnosis and on the 30th day of remission-induction therapy. Laboratory parameters were analyzed in both the AML and control groups, including white blood cell (WBC) and platelet counts, hemoglobin (Hb), creatinine, uric acid, and lactate dehydrogenase levels. Additionally, PERK pathway–related markers [eukaryotic translation initiation factor 2-alpha kinase 3 (eIF2AK3), glucose-regulated protein 78 (GRP78), and CCAAT/enhancer-binding protein homologous protein (CHOP)], the apoptosis-related caspase 3, and the hypoxia-inducible factor-1 alpha (HIF-1α) were measured.

Venous blood samples from both AML patients and healthy controls were collected in hemogram tubes containing sodium ethylenediaminetetraacetic acid (Na-EDTA). Following centrifugation at 1800 rpm for 10 min, plasma samples were transferred into microvolume capped tubes and stored at −80 °C. On the day of analysis, plasma samples stored at −80 °C were thawed at room temperature prior to biochemical assessment. Thawed samples were homogenized using a vortex mixer, and biochemical parameters were subsequently measured.

HIF-1α, eIF2AK3, GRP78, ATF6, CHOP, and caspase 3 levels were determined using enzyme-linked immunosorbent assay (ELISA) kits. HIF-1α, eIF2AK3, and GRP78 concentrations were expressed in ng/mL, whereas ATF6, CHOP, and caspase 3 concentrations were expressed in pg/mL.

### 2.3. Statistical analysis

Data were analyzed using SPSS Statistics version 25.0 (IBM Corp., Armonk, NY, USA). Descriptive statistics were presented as frequencies and percentages for categorical variables, and as median (minimum–maximum) or mean ± standard deviation (SD) for continuous variables. The normality of data distribution within groups was assessed using the Shapiro–Wilk test. Student’s t-test was applied for normally distributed continuous variables, whereas the Mann–Whitney U-test was used for nonparametric data. The chi-square test was used to analyze categorical variables. The Wilcoxon signed-rank test was used to evaluate changes between paired or dependent groups. The Spearman rank correlation test was used to assess correlations between variables. The Kaplan–Meier method was used to estimate OS and relapse-free survival (RFS). Cox proportional hazards regression analysis was performed to identify prognostic factors influencing survival outcomes. Statistical significance was set at p < 0.05.

## 3. Results

### 3.1. Patients

In this study, a total of 40 participants were included, comprising 19 (47%) patients with AML and 21 (53%) healthy controls. Among all participants, 17 (43%) were male and 23 (58%) were female, with a median age of 50 years (range: 20–65) and a mean age of 49 ± 10 years. In the AML group, six (32%) patients were classified as low risk, 10 (53%) as intermediate risk, and three (16%) as high risk. FLT3 and nucleophosmin (NPM1) mutations were detected in three (15%) and two (11%) patients, respectively, while no mutation was identified in 14 (74%) patients. Cytogenetic abnormalities t(15;17), t(8;21), and t(16;16) were identified in four (21%), one (5%), and one (5%) patients, respectively, whereas no cytogenetic abnormality was detected in 13 (69%) patients.

Platelet count and Hb levels were significantly lower in the AML group than in the control group, whereas no significant difference was observed in WBC count (p < 0.001, p < 0.001, and p = 0.688, respectively). The demographic and laboratory characteristics of the AML and control groups are summarized in Table 1.

### 3.2. Endoplasmic reticulum stress, hypoxia, and apoptosis marker levels at diagnosis

HIF-1α, eIF2AK3, GRP78, ATF6, CHOP, and caspase 3 levels were significantly higher in the AML group than in the control group (p = 0.019, 0.005, <0.001, 0.006, <0.001, and <0.001, respectively) (Table 2).

### 3.3. Changes in endoplasmic reticulum stress, hypoxia, and apoptosis marker levels following treatment

No significant differences were observed in HIF-1α, GRP78, ATF6, CHOP, or caspase 3 levels between diagnosis and the 30th day of remission-induction therapy in the AML group, whereas a significant decrease was observed in eIF2AK3 levels (p = 0.808, 0.698, 0.917, 0.349, 0.972, and 0.049, respectively) (Table 3).

Following remission-induction therapy, 16 (84%) patients achieved complete remission, whereas three (16%) were classified as primary refractory. Among the patients who achieved remission, relapse was observed in seven (44%), whereas nine (56%) remained relapse free. Overall, 10 (53%) patients were classified as relapsed or refractory, and nine (47%) remained in remission. No significant differences were detected in HIF-1α, eIF2AK3, GRP78, ATF6, CHOP, or caspase 3 levels between relapsed/refractory and remission groups (p = 0.465, 0.133, 0.720, 0.278, 0.842, and 0.182, respectively) (Table 4).

### 3.4. Relationship between endoplasmic reticulum stress, hypoxia, and apoptosis marker levels and survival

At the end of the follow-up period, four (21%) patients had died, whereas 15 (79%) were alive. The 12-month OS rate was 79%, and the 12-month RFS rate was 69%. The mean OS was 14.3 months (95% CI: 11.4–17.2), and the mean RFS was 12.4 months (95% CI: 9.6–15.3).

In the Cox proportional hazards regression analysis performed in the AML group, no significant association was observed between OS and age, risk category, HIF-1α, eIF2AK3, GRP78, ATF6, CHOP, or caspase 3 levels [Exp (B) for age = 0.714, p = 0.721; risk = 0.000, p = 0.781; HIF-1α = 0.000, p = 0.500; eIF2AK3 = 0.077, p = 0.223; GRP78 = 474, p = 0.947; ATF6 = 1.007, p = 0.569; CHOP = 1.036, p = 0.667; caspase 3 = 1.119, p = 0.804; Omnibus chi-square = 21.904, df = 9, p = 0.009].

## 4. Discussion

ER is a crucial organelle that regulates intracellular processes such as lipid metabolism, calcium homeostasis, protein synthesis and folding, and postranslational modification of transmembrane and secretory proteins. When ER homeostasis is disrupted due to various factors, misfolded or unfolded proteins accumulate within the ER, a condition known as ERS. In response to ERS, the UPR, which involves PERK, ATF6, and IRE1α signaling pathways, is activated [8]. Under normal physiological conditions, PERK, ATF6, and IRE1α signaling pathways remain inactive by binding to GRP78 at the ER membrane. When the misfolded protein load increases in the ER, GRP78 dissociates from these sensors and translocates to the ER lumen to facilitate protein folding, leading to the activation of PERK, ATF6, and IRE1α pathways [7,8,12].

The uncontrolled proliferation of tumor cells and inadequate vascularization result in a limited supply of oxygen and nutrients. The rapid cellular proliferation leads to excessive accumulation of unfolded or misfolded proteins within the ER. Collectively, these conditions contribute to the development of ERS and activation of the UPR. Persistent activation of the UPR has been associated with tumor cell proliferation, disease progression, and metastasis [13]. Several studies have reported increased UPR activity in various hematologic malignancies [14–19]. Rapid proliferation of leukemic stem cells (LSCs) leads to the accumulation of metabolic by-products and increased production of reactive oxygen species. This process promotes the accumulation of unfolded or misfolded proteins within these cells. Additionally, calreticulin produced through activation of the ATF6 signaling pathway inhibits myeloid differentiation by suppressing CEBPA translation [20]. In a study involving AML patients, UPR activation was detected in 25% of cases, with elevated sXBP1 levels and marked overexpression of the ER chaperone calreticulin [21]. In an in vitro study by Zhou et al., Jun expression—a direct regulator of UPR target genes—was found to be significantly increased in AML cells. Consequently, Jun was shown to enhance ERS in AML cells by directly binding to the promoters of XBP1, ATF4, and CHOP [22]. In the present study, GRP78, eIF2AK3, ATF6, and CHOP levels were significantly higher in the AML group than in the control group. In conclusion, our findings demonstrate that the UPR is activated in patients with AML. Infection and inflammation can trigger ERS by increasing cytokine levels such as IL-6 and TNF-α. Consequently, this process results in enhanced PERK and ATF6 activity. Although antibiotics typically suppress infection and may attenuate the UPR response, certain antibiotics can paradoxically enhance PERK and ATF6 activity. Additionally, anemia-induced hypoxia may also contribute to increased PERK and ATF6 activity. Therefore, these factors should be considered as potential confounders influencing the levels of markers associated with PERK and ATF6 [7].

Several studies have evaluated the relationship between therapeutic interventions and ERS in patients with AML. Hua et al. demonstrated that metformin enhanced sensitivity to venetoclax through the ERS-related CHOP signaling pathway in AML cell lines [23]. Li et al. reported that inhibition of DNAJC10 enhanced ERS and activated the PERK–eIF2α–ATF4 signaling pathway. This inhibition consequently enhanced the chemotherapeutic efficacy of cytarabine (Ara-C) and daunorubicin in leukemic cells [24]. These findings suggest that induction of ER stress and subsequent UPR activation may promote the elimination of AML cells. In the present study, GRP78, ATF6, and CHOP levels in AML patients did not decrease following Ara-C and idarubicin treatment, whereas eIF2AK3 levels significantly decreased after therapy.

A gene-expression profiling study on AML and ERS demonstrated that patients with a low ERS-related prognostic risk score had higher survival rates than those with a high risk score [25]. Similarly, a bioinformatics analysis of 42 ERS-related genes classified AML patients into low- and high-risk groups, revealing longer survival in the low-risk group. Collectively, these studies suggest that risk assessment based on ER stress–related gene expression profiles could serve as a prognostic indicator in AML [26]. However, in our cohort, no significant differences were observed in GRP78, eIF2AK3, ATF6, or CHOP levels at diagnosis between treatment-responsive and nonresponsive patients. Furthermore, no significant associations were found between ERS marker levels and overall survival.

Hypoxia is a key physiological stressor that stimulates ERS and activates the UPR. Under hypoxic conditions, activation of GRP78, a target gene regulated by HIF-1α, contributes to the activation of the UPR [27]. Ijabi et al. reported higher HIF-1α and GRP78 levels in patients with chronic lymphocytic leukemia compared to healthy controls [28]. In the present study, significant differences were observed between the AML and control groups in Hb, HIF-1α, GRP78, and other ERS markers. In conclusion, our findings suggest that in AML, both anemia and hypoxia resulting from rapid cellular proliferation contribute to ERS activation.

Studies on hematologic malignancies have demonstrated correlations between caspase 3 and other ERS markers, particularly CHOP [29,30]. Similarly, our study demonstrated higher caspase 3 and ERS marker levels in the AML group than in the control group. Moreover, a positive correlation was identified between caspase 3 and CHOP levels, whereas no significant correlations were observed among the other ERS markers. In conclusion, these findings support the association between CHOP expression and apoptosis in AML.

## 5. Conclusion

The PERK and ATF6 signaling pathways were found to be activated in patients with AML. Targeted therapies modulating the UPR may represent promising therapeutic strategies for patients with AML.

## 6. Limitations

The primary limitation of this study is the relatively small sample size. Additionally, the inability to evaluate some patients on the 30th day of treatment constitutes another important limitation of the study.

## Figures and Tables

**Table 1:** Demographic and laboratory characteristics of patients with AML and healthy control groups.

Characteristics	AML group (n = 19)	Control group (n = 21)	p-value

Age years, median (min–max), mean (±SD)	54 (20–65), 50 (13)	49 (39–56), 49 (6)	0.187

Sex, n (%)			
Female	8 (35)	15 (65)	0.061
Male	11 (65)	6 (35)	

WBC ×10^9^/L, median (min–max), mean (±SD)	7.4 (1.6–299), 56.5 (86.4)	6.1 (3.1–9.4), 6.2 (1.6)	0.688

Platelet ×10^9^/L, median (min–max), mean (±SD)	40 (10–175), 65.8 (47.9)	252 (178–437), 263.8 (65.5)	<0.001*

Hemoglobin g/dL, median (min–max), mean (±SD)	9.4 (6.1–13.9), 9.6 (1.9)	13.8 (11.6–16.4), 13.7 (1.3)	<0.001*

AML: acute myeloid leukemia; SD: standard deviation; WBC: white blood cell.

* p < 0.05 was considered statistically significant.

Age, WBC, and platelet data were analyzed using nonparametric methods, whereas hemoglobin data were analyzed using a parametric method.

**Table 2:** Endoplasmic reticulum stress, hypoxia, and apoptosis marker levels at diagnosis.

Markers	AML group (n = 19)	Control group (n = 21)	p-value
HIF-1α ng/mL, median (min–max), mean (±SD)	0.76 (0.58–1.17), 0.78 (0.14)	0.73 (0.59–0.93), 0.74 (0.10)	0.019*
eIF2AK3 ng/mL, median (min–max), mean (±SD)	4.46 (2.12–16.9), 5.73 (3.72)	3.55 (2.12–9.02), 4.15 (1.56)	0.005*
GRP78 ng/mL, median (min–max), mean (±SD)	1.24 (0.80–8.06), 1.77 (1.50)	1.01 (0.80–2.08), 1.09 (0.28)	<0.001*
ATF6 pg/mL, median (min–max), mean (±SD)	577.56 (364.76–2833.49), 699.29 (500.79)	532.51 (364.76–664.30), 523.59 (80.32)	0.006*
CHOP pg/mL, median (min–max), mean (±SD)	310.45 (221.31–1165.54), 360.41 (177.25)	281.28 (221.31–358.85), 281.47 (38.91)	<0.001*
Kaspaz 3 pg/mL, median (min–max), mean (±SD)	3.71 (2.08–56.27), 5.61 (8.62)	3.40 (2.08–4.27), 3.34 (0.57)	<0.001*

AML: acute myeloid leukemia; HIF-1α: hypoxia-inducible factor-1 alpha; SD: standard deviation; eIF2AK3: eukaryotic translation initiation factor 2-alpha kinase 3; GRP78: glucose-regulated protein 78; ATF6: activating transcription factor 6; CHOP: CCAAT/enhancer-binding protein homologous protein.

* p < 0.05 was considered statistically significant.

eIF2AK3, GRP78, ATF6, CHOP, and caspase 3 data were analyzed using nonparametric methods, whereas HIF-1α data were analyzed using a parametric method.

**Table 3:** Changes in endoplasmic reticulum stress, hypoxia, and apoptosis marker levels following treatment.

Markers	The first day of remission-induction (n = 19)	The 30th day of remission-induction (n = 13)	p-value
HIF-1α ng/mL, median (min–max), mean (±SD)	0.76 (0.58–1.17), 0.78 (0.14)	0.87 (0.62–1.12), 0.88 (0.16)	0.808
eIF2AK3 ng/mL, median (min–max), mean (±SD)	4.46 (2.12–16.9), 5.73 (3.72)	4.14 (3.52–7.23), 4.60 (1.13)	0.049*
GRP78 ng/mL, median (min–max), mean (±SD)	1.24 (0.80–8.06), 1.77 (1.50)	1.35 (0.75–2.41), 1.46 (0.48)	0.698
ATF6 pg/mL, median (min–max), mean (±SD)	577.56 (364.76–2833.49), 699.29 (500.79)	733.61 (400.61–4412.73), 1092 (1051.91)	0.917
CHOP pg/mL, median (min–max), mean (±SD)	310.45 (221.31–1165.54), 360.41 (177.25)	317.98 (256.20–435.06), 324.85 (57.52)	0.349
Kaspaz 3 pg/mL, median (min–max), mean (±SD)	3.71 (2.08–56.27), 5.61 (8.62)	3.49 (2.90–5.31), 3.86 (0.78)	0.972

HIF-1α: hypoxia-inducible factor-1 alpha; SD: standard deviation; eIF2AK3: eukaryotic translation initiation factor 2-alpha kinase 3; GRP78: glucose-regulated protein 78; ATF6: activating transcription factor 6; CHOP: CCAAT/enhancer-binding protein homologous protein.

* p < 0.05 was considered statistically significant.

ATF6 and caspase 3 data were analyzed using nonparametric methods, whereas HIF-1α, eIF2AK3, GRP78, and CHOP data were analyzed using parametric methods.

**Table 4:** Relationship between endoplasmic reticulum stress, hypoxia, and apoptosis markers with treatment response status.

Markers	Response (n = 9)	No response (n = 10)	p-value
HIF-1α ng/mL, median (min–max), mean (±SD)	0.83 (0.53–1.17), 0.86 (0.19)	0.79 (0.66–1.06), 0.81 (0.12)	0.465
eIF2AK3 ng/mL, median (min–max), mean (±SD)	7.10 (3.24–16.97), 8.93 (4.93)	4.80 (2.52–15.49), 6.19 (4.07)	0.133
GRP78 ng/mL, median (min–max), mean (±SD)	1.56 (1.11–8.06), 2.37 (2.20)	1.61 (1.14–5.35), 2.65 (1.73)	0.720
ATF6 pg/mL, median (min–max), mean (±SD)	754.06 (437.47–2600.73), 953.03 (651.12)	577.85 (410.71–2833.49), 839.91 (732.94)	0.278
CHOP pg/mL, median (min–max), mean (±SD)	366.62 (307.32–1165.54), 451.97 (270.10)	370.44 (271.32–846.20), 443.77 (193.10)	0.842
Caspase 3 pg/mL, median (min–max), mean (±SD)	4.19 (2.79–56.27), 9.64 (17.50)	5.48 (3.63–18.36), 6.76 (4.42)	0.182

HIF-1α: hypoxia-inducible factor-1 alpha; SD: standard deviation; eIF2AK3: eukaryotic translation initiation factor 2-alpha kinase 3; GRP78: glucose-regulated protein 78; ATF6: activating transcription factor 6; CHOP: CCAAT/enhancer-binding protein homologous protein.

* p < 0.05 was considered statistically significant.

eIF2AK3, GRP78, ATF6, CHOP, and caspase 3 data were analyzed using nonparametric methods, whereas HIF-1α data were analyzed using a parametric method.
